# Evaluation of an Injectable Biphasic Calcium Sulfate/Hydroxyapatite Cement for the Augmentation of Fenestrated Pedicle Screws in Osteoporotic Vertebrae: A Biomechanical Cadaver Study

**DOI:** 10.3390/jfb13040269

**Published:** 2022-12-01

**Authors:** Xinggui Tian, Deepak B. Raina, Corina Vater, David Kilian, Tilman Ahlfeld, Ivan Platzek, Ute Nimtschke, Magnus Tägil, Lars Lidgren, Alexander Thomas, Uwe Platz, Klaus-Dieter Schaser, Alexander C. Disch, Stefan Zwingenberger

**Affiliations:** 1 University Center of Orthopaedic, Trauma and Plastic Surgery, University Hospital Carl Gustav Carus at Technische Universität Dresden, 01307 Dresden, Germany; 2 Center for Translational Bone, Joint and Soft Tissue Research, Faculty of Medicine, University Hospital Carl Gustav Carus at Technische Universität Dresden, 01307 Dresden, Germany; 3 Department of Clinical Sciences Lund, Faculty of Medicine, Lund University, 22184 Lund, Sweden; 4 Department of Radiology, University Hospital Carl Gustav Carus at Technische Universität Dresden, 01307 Dresden, Germany; 5 Institute of Anatomy, University Hospital Carl Gustav Carus at Technische Universität Dresden, 01307 Dresden, Germany

**Keywords:** biomaterial, biomechanical, calcium sulfate/hydroxyapatite, cement, osteoporosis, pedicle screw augmentation

## Abstract

Cement augmentation of pedicle screws is one of the most promising approaches to enhance the anchoring of screws in the osteoporotic spine. To date, there is no ideal cement for pedicle screw augmentation. The purpose of this study was to investigate whether an injectable, bioactive, and degradable calcium sulfate/hydroxyapatite (CaS/HA) cement could increase the maximum pull-out force of pedicle screws in osteoporotic vertebrae. Herein, 17 osteoporotic thoracic and lumbar vertebrae were obtained from a single fresh-frozen human cadaver and instrumented with fenestrated pedicle screws. The right screw in each vertebra was augmented with CaS/HA cement and the un-augmented left side served as a paired control. The cement distribution, interdigitation ability, and cement leakage were evaluated using radiographs. Furthermore, pull-out testing was used to evaluate the immediate mechanical effect of CaS/HA augmentation on the pedicle screws. The CaS/HA cement presented good distribution and interdigitation ability without leakage into the spinal canal. Augmentation significantly enhanced the maximum pull-out force of the pedicle screw in which the augmented side was 39.0% higher than the pedicle-screw-alone side. Therefore, the novel biodegradable biphasic CaS/HA cement could be a promising material for pedicle screw augmentation in the osteoporotic spine.

## 1. Introduction

Osteoporosis is the most common age-related bone disease. Over 200 million people worldwide suffer from osteoporosis [1]. The prevalence of osteoporosis has been continuously escalating with increasingly elderly populations, with more than 70% of people over the age of 80 being affected [2]. With increased life expectancy, improved quality of life, and the desire to maintain physical activity in older adults, the need for spinal surgery in elderly patients suffering from osteoporosis has increased accordingly [3]. The prevalence of osteoporosis in men older than 50 and postmenopausal women when undergoing spinal surgery was reported as 14.5% and 51.3%, respectively, by Xie et al. [4]. Pedicle screws are the most commonly used implants to achieve posterior fixation of the lumbar and thoracic spine. The key to an adequate base for a stable fixation is the bony purchase of the pedicle screws in the pedicle and trabecular bone of the vertebral body [5]. The specific morphology of the trabecular meshwork affects the mechanical grasp of the screw and, therefore, influences the screw integration at the bone–metal interface [6]. In osteoporotic vertebrae, bone-metal osteointegration is significantly reduced which often leads to the loosening of the screw [6] with a rate of up to 62% [7].

Over the past few decades, numerous techniques have been developed to improve the anchorage of pedicle screws in order to reduce the risk of screw failure. These techniques primarily suggested larger outer diameters or an extended length of screws, screws with different thread profiles, a cylindrical or conical core shape, expanding screws, bi-cortical pedicle screws, coated pedicle screws, or cement augmentation screws [8,9]. The cement-augmented pedicle screw technique is considered one of the most promising approaches for the improvement of screw fixation. A recent meta-analysis showed that screw augmentation significantly reduced the loosening rate by a factor of 10 (non-augmentation screws vs. augmentation screws: 22.5% vs. 2.2%) [10]. To date, polymethyl methacrylate (PMMA) is the only material that has been clinically approved by the FDA to augment pedicle screw fixation. Due to its high viscosity and injectability, PMMA is advantageous for augmenting pedicle screws in the osteoporotic bone with a high stability. However, PMMA also has some drawbacks limiting its clinical application. PMMA is a rather stiff, high-strength epoxy resin with a higher mechanical strength than osteoporotic bone. This biomechanical mismatch leads to a shielding effect on the adjacent vertebrae or disc [11]. The strong exothermic reaction during PMMA polymerization can further damage the surrounding already-fragile bone, which weakens the fixation between the bone and cement and can finally lead to aseptic loosening [1]. Most importantly, due to its non-degradability and lack of bioactivity, PMMA neither forms a stable bio-bonding with the bone nor induces bone ingrowth or osseointegration [12]. Due to its non-resorbability, a chronic inflammatory response may occur, further compromising the strength and integrity of the implant [13]. PMMA excessively adheres to the screws, making it difficult to remove the screw during revision surgery [12]. In addition, its monomer component methyl methacrylate (MMA) is known to be toxic to healthy organs [9]. These PMMA-associated drawbacks have created a need for bioactive or bioresorbable functionalized cements to achieve better pedicle screw augmentation in osteoporotic bone. Calcium phosphate [9] as well as calcium sulfate cements [14] with high biodegradability were developed as PMMA-replacing alternative candidates for pedicle screw augmentation. However, the long curing time of calcium phosphate cements [15] and the rapid degradation rate of calcium sulfate cements [16] limit their clinical application.

In this study, we aim to show that a recently established injectable calcium sulfate/hydroxyapatite (CaS/HA) biomaterial, which has been approved for human use both in Europe and North America [17], may meet the requirements of pedicle screw augmentation for improved efficacy. The CaS/HA cement is a powder-based biomaterial that forms a paste when mixed with the liquid phase, with proper biocompatibility [18]. It can be injected for about 5 min, molded for up to 9 min after mixing, and is transformed into a solid mass approximately 15 min after initiating the mixing procedure [19]. The compressive strength of the material is higher compared to cancellous bone [20]. Clinical studies have shown that CaS/HA cement as a substitute for PMMA has achieved good clinical results in the treatment of osteoporotic vertebral fractures through percutaneous vertebroplasty [21,22], providing strong evidence that CaS/HA cement can be used to treat osteoporotic spinal diseases. Previous preclinical studies have shown that CaS/HA can enhance screw anchorage in native or synthetic bones [23,24]. Our latest study also showed that CaS/HA-enhanced lag-screw anchorage with high extraction forces is comparable to PMMA in cadaveric femoral heads as well as in patients undergoing treatment for intertrochanteric fractures [25]. In addition, CaS/HA cement is a degradable material that can serve as a scaffold for new bone ingrowth in order to achieve better osseointegration. Its isothermal setting reaction will be compatible with surrounding tissues as it does not lead to thermally induced necrosis [25]. Thus, the purpose of this study was to investigate whether degradable CaS/HA cement could be used for pedicle screw augmentation in osteoporotic vertebrae.

We hypothesized that CaS/HA cement could strengthen the interface between the pedicle screw and the vertebral trabecular bone with the potential to increase the pull-out force of the pedicle screw in the osteoporotic vertebrae in a preliminary proof-of-principle study.

## 2. Materials and Methods

### 2.1. Methodology

**Figure 1:**
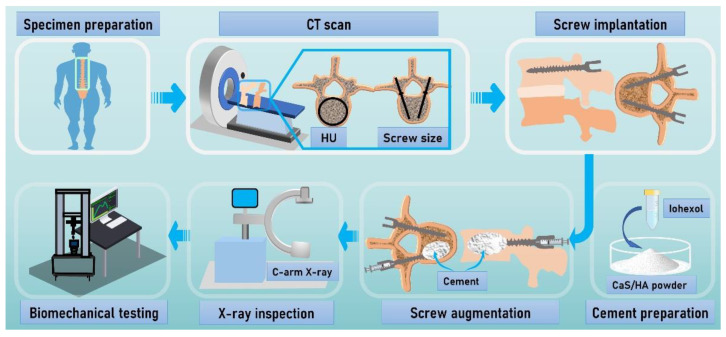
Flow chart summarizing the methodology. After the specimens’ acquisition, computed tomography (CT) scan was performed to determine that the specimen met the experimental criteria and to determine the parameters of the pedicle screws. Pedicle screws were implanted and augmented with CaS/HA cement, and then a pull-out experiment was performed to evaluate the effect of the augmentation. Abbreviations: HU: Hounsfield units; CaS/HA: calcium sulfate/hydroxyapatite.

A male human cadaver was used in this study as a model of osteoporotic vertebrae to test the hypothesis that the CaS/HA cement could augment the pedicle screws with a potential improvement of the immediate anchorage in the osteoporotic vertebrae. The computed tomography (CT) scan was performed to measure the Hounsfield Units (HU). The fenestrated pedicle screws were implanted into the osteoporotic vertebrae, in which the right side of the vertebra was augmented with the CaS/HA cement and the left side of the vertebra was not augmented. During the procedure, the C-arm X-ray was used to evaluate cement distribution and cement leakage. After the pedicle screw augmentation, the specimens were embedded in epoxy resin for pull-out testing to evaluate the pull-out force. The maximum pull-out force was used to quantify the effect of cement augmentation by comparison with the non-augmented side. (Figure 1). 

### 2.2. Specimen Preparation

A total of 17 vertebrae (12 thoracic and 5 lumbar) were obtained from a fresh-frozen cadaver of an 81-year-old male (Institute of Anatomy, University Hospital Carl Gustav Carus at Technische Universität Dresden, Dresden, Germany). All soft tissues on the vertebral body were completely removed and CT scans of all vertebral bodies were performed (Department of Radiology, University Hospital Carl Gustav Carus at Technische Universität Dresden). The diameter of the pedicle and the length of the vertebral body were measured to determine the respective size of each pedicle screw. The radiodensity in Hounsfield Units (HU) of the vertebral body, screw trajectory, and augmentation area were also measured using AGFA HealthCare software (AGFA; Mortsel, Belgium). The HU of vertebral bodies was measured according to a method described by Li et al. [26], and the HU of screw trajectory was measured using a method previously described by Zhang et al. [27]. In addition, a circular area with a diameter of 8 mm at the front end of the screw trajectory was defined as the ROI of the potentially augmented area in this study. 

### 2.3. Pedicle Screw Implantation

The cortex and hyperplastic osteophytes of the lamina were removed with a rongeur, so that the polyaxial tulip-head of the pedicle screw could be rotated freely to prevent it from blocking the pedicle screw. A pilot hole was made by an awl oriented forward and inward along the pedicle axis. Then we checked the hole with a probe to ensure that the pedicle was not damaged. Each hole was tapped using a 4.5 mm tap, then each fenestrated pedicle screw (uCentum, Ulrich Medical, Ulm, Germany) was implanted with a ratchet screwdriver using a free-hand technique until the hub of the screw had been firmly fixed on the posterior cortex. Because the trajectory of the screw during implantation affects the required extraction force later, the classical trajectory of passing the center of the pedicle parallel to the superior and inferior endplates was adopted in this study [28]. All instrumentation was completed by one experienced spine surgeon. The specimens were stored in sealed vacuum bags at −20 °C until further preparation and testing.

### 2.4. CaS/HA Cement Preparation and Augmentation Procedure

According to the preparation protocol, the CaS/HA cement (Cerament, Bone Support AB, Lund, Sweden), which consists of a premixed powder of 60 weight% (wt%) CaS and 40 wt% HA, was mixed with iohexol (1 g CaS/HA:0.43 mL iohexol) in a 6-well plate for 30 s. The CaS/HA slurry was then transferred into the syringe used for injection. This process allowed the cement to cure for 4 min prior to injection [17,19]. During this period, the syringe was connected to the tail of the fenestrated pedicle screw through a special metal tube. Then the CaS/HA paste was injected into the right-side screws. A total of 1 mL CaS/HA paste was used for each thoracic vertebra and 2 mL was used for each lumbar vertebra. During injection, C-arm fluoroscopy radiographs were taken to evaluate cement distribution, interdigitation ability, and cement leakage. The CaS/HA cement was allowed to harden at room temperature for 24 h before mechanical testing. 

### 2.5. Mechanical Testing 

**Figure 2:**
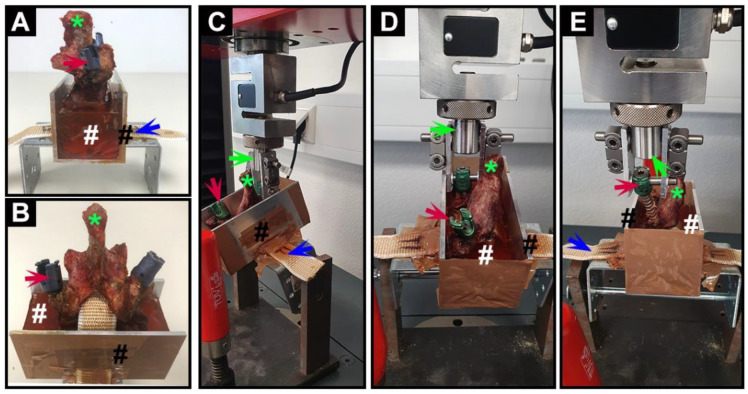
Photographs of vertebra fixation and biomechanical testing process. (**A**) Lateral view and (**B**) anterior view of a vertebra (*) fixed in the customized metal container (black #) filled with epoxy resin (white #); (**C**) lateral view; and (**D**) anterior view of the mechanical testing process of pedicle screws; and (**E**) the picture of pedicle screws being successfully pulled out from the vertebra. Red arrows indicate the pedicle screws; blue arrows indicate the fixed band; green arrows indicate the piston of the testing machine.

A customized metal container was used to hold the vertebra, while a band was used to fix the vertebra by insertion through the spinal canal. The vertebrae were then embedded in epoxy resin (Yachticon, Norderstedt, Germany) with the embedded line of each vertebra under the pedicle to avoid interference with mechanical testing (Figure 2A,B). This process required setting overnight at room temperature to obtain an exact, stable fixation. For the pull-out test, the customized container was fixed on the bottom of the testing machine (Z010, Zwick-Roell, Ulm, Germany) with a titanium rod. In this position, it could be freely adjusted in the sagittal plain of the pedicle to ensure that the implantation direction of each pedicle screw was consistent with the direction of the piston of the machine. In this case, the pedicle screw was securely attached to the piston with a nut. In addition to the pull-out force along the longitudinal axis of the screw, this procedure should avoid interference with additional forces in order to allow reliable quantification of the shear stability of the screw-bone interface (Figure 2C,D). The screws were pulled out at a displacement of 10 mm/min using a custom-made jig on the universal testing machine in displacement-controlled mode (Figure 2E). To avoid any biased tendency in the results by the test order, the tests were alternated between right (cement-augmented) and left (non-augmented) sides. The pull-out strength of a pedicle screw was defined as the point at which the force peaks in the force-displacement diagram in newtons (N). Stiffness was defined as the slope in the linear region of the force-displacement curve.

### 2.6. Statistics

Data were presented as mean ± standard deviation (SD). Statistical analysis was performed by using GraphPad Prism 8 (GraphPad Software, San Diego, CA, USA). Significant differences between the groups were evaluated using the paired t-test. The level of significance was chosen at *p* < 0.05 (*).

### 2.7. Ethical Approval

This study involved cadaveric specimens voluntarily donated by a donor to the Institute of Anatomy, University Hospital Carl Gustav Carus at Technische Universität Dresden for the advancement of science. This study has been carried out in accordance with the Code of Ethics of the World Medical Association (WMA Statement on Organ and Tissue Donation) and the local ethics policy of Dresden University Hospital for experiments.

## 3. Results

### 3.1. Specimen Preparation

**Figure 3:**
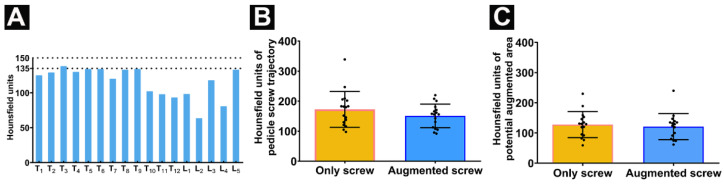
Quantification of radiodensity in CT scans: (**A**) Radiodensity in Hounsfield units (HU) for all vertebral bodies T1–T12 and L1–L5; (**B**) Radiodensity values (in HU) of pedicle screw trajectory comparing augmented screw side and only screw side; and (**C**) the HU between the potential augmented area and contralateral area. Data are presented as mean ± SD. The two horizontal dotted lines in panel A represent the critical values for diagnosing osteoporosis by HU in previous literature [29].

For this cadaveric study, all thoracic and lumbar vertebrae from one cadaveric donor were collected. The T7 height was reduced which may have suffered a previous osteoporotic vertebral compression fracture, without previous cement-related surgical treatment. The applied individual, vertebra-specific pedicle screw sizes are listed in Table 1. The radiodensity of all vertebral bodies was below 150 HU while the HU values of all vertebral bodies except T3 (138) were also detected as lower than 135 (Figure 3A). No significant difference was detected comparing the HU of the pedicle screw trajectory between the potentially cement-augmented (right) side and the un-augmented (left) side (Figure 3B) and the HU between the potential augmented area and contralateral relative area (Figure 3C).

### 3.2. Pedicle Screw Implantation 

**Figure 4:**
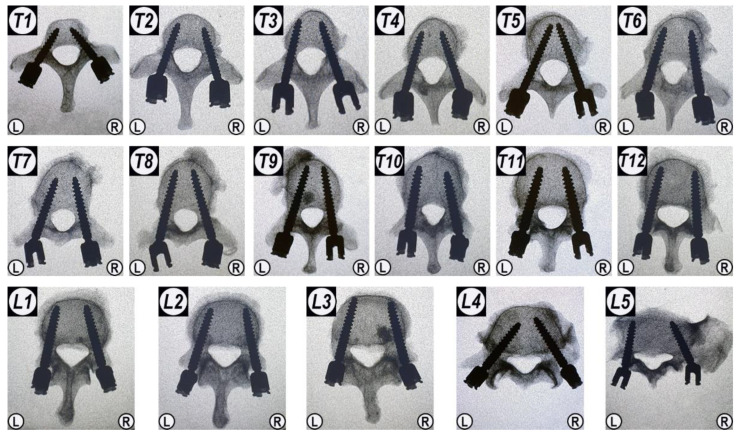
The anteroposterior X-ray images of all vertebrae after the fenestrated pedicle screw implantation prior to augmentation with CaS/HA material. “L” and “R” indicate the left and right side; T1–T12 and L1–L5 indicate the thoracic and lumbar position of the vertebrae.

All pedicle screws were successfully implanted into the vertebrae. After pedicle screw implantation, X-ray images were taken in order to demonstrate that the placement of all pedicle screws was accurate, and that no pedicle or vertebral body was damaged during the operation (Figure 4).

### 3.3. Trajectories of Augmented and Non-Augmented Pedicle Screws

**Figure 5:**
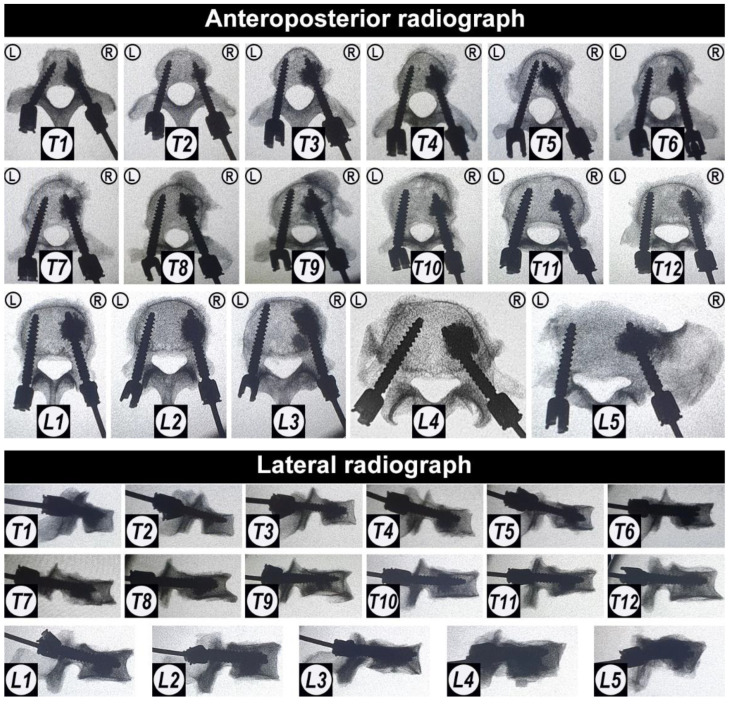
The anteroposterior radiograph and the lateral radiograph of the fenestrated pedicle screws augmented with CaS/HA cement. “L” and “R” indicate the left and right side; T1–T12 and L1–L5 indicate the thoracic and lumbar position of the vertebrae.

The CaS/HA cement was completely and successfully injected in all vertebrae (1 mL in the thoracic vertebrae and 2 mL in the lumbar vertebrae) under fluoroscopic guidance. After cement injection, X-ray images demonstrated that the CaS/HA cement was evenly distributed around the anterior hole of the fenestrated pedicle screws both in the coronal plane from the anteroposterior radiograph and in the sagittal plane from the lateral radiograph in all vertebrae. No obvious cement leakage was seen into the spinal canal. In addition, the irregular shape of the cement edges also indicated the adequate cement-bone interdigitation ability in the trabecular bone (Figure 5).

**Figure 6:**
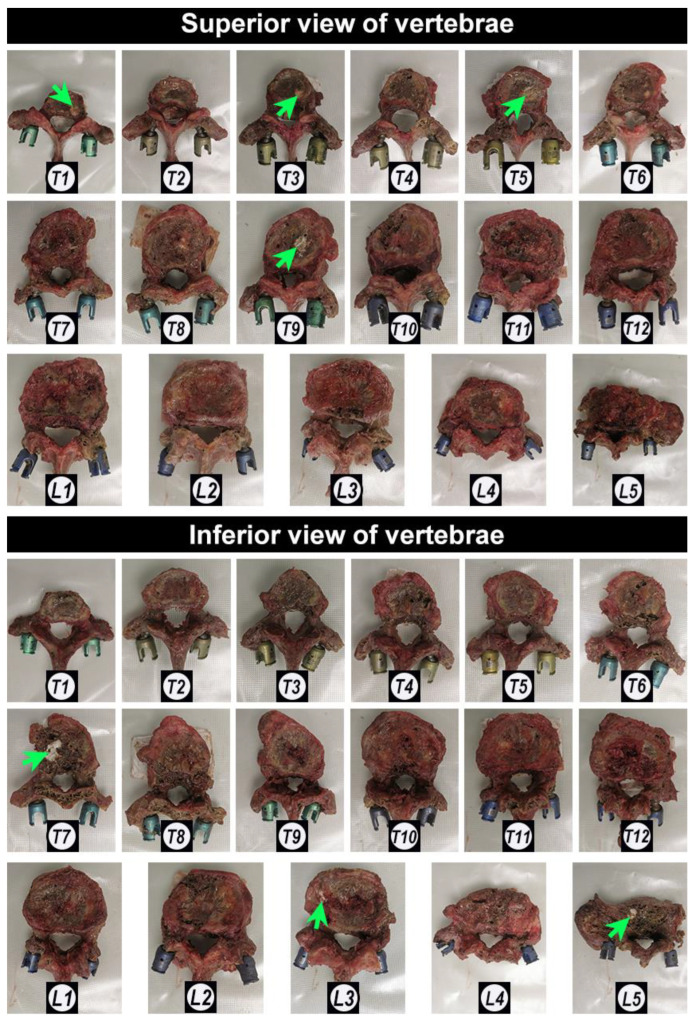
Photographs of the appearance of all the vertebrae after cement injection. Green arrows indicate the CaS/HA cement. T1–T12 and L1–L5 indicate the thoracic and lumbar position of the vertebrae.

Further visual observation of the vertebral appearance after cement injection revealed that the cement was exposed in the superior endplate plane of four vertebrae (T1, T3, T5, and T9) and in the inferior endplate plane of three vertebrae (T7, L3, and L5) (Figure 6).

### 3.4. CaS/HA Augmentation Leads to an Increased Pull-Out Force

**Figure 7:**
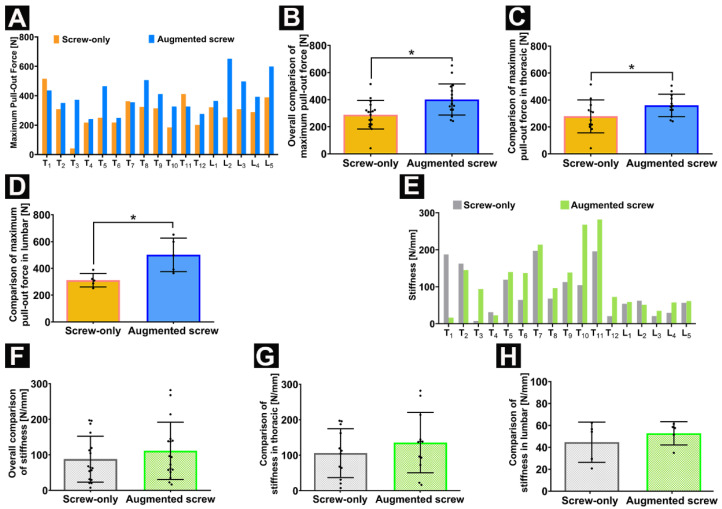
(**A**–**D**) The maximum pull-out force in N for screws pulled out from the cement-augmented screw side and screw-only side; (**A**,**B**) overall comparison; (**C**) comparison for thoracic vertebrae; and (**D**) lumbar comparison of screw only vs. augmented screw. (**E**–**H**) Stiffness in N/mm of vertebrae comparison between the cement-augmented screw side and screw-only side; (**E**,**F**) overall; (**G**) thoracic; and (**H**) lumbar comparison. Data are presented as mean ± SD, * *p* < 0.05.

All specimens were stabilized in the customized metal container to minimize variation. Regarding the maximum pull-out force, the determined force for the augmented screw was higher than the pull-out force of the pedicle screw without augmentation among 14 vertebrae. Whereas test outliers on the unenhanced side of T3 may be due to the destruction of the trabecular microstructure of the pedicle and vertebral body, cement augmentation significantly increased its pull-out resistance although the pedicle trabecular microarchitecture on the augmented side was also destroyed from the analysis of CT scans (Dragonfly, ORS Inc., Montreal, QC, Canada) (Supplementary Materials, Figure S1A). The maximum pull-out force of the screw on the contralateral side of three vertebrae was slightly higher than on the augmentation side. Each individual pull-out force of the augmentation side was lower than the corresponding pedicle screw-alone side occurring in the thoracic spine (T1, T7, and T11). From the analysis of the CT scans, it was found that the reason for these undesired results may be related to the microfracture of the right part of the vertebral body at T7 and T11 and the obvious cortical bone hyperplasia of the left pedicle at T1 detected by comparison with the right side (Supplementary Materials, Figure S1B–D). In the lumbar spine, all the pull-out force figures of the augmentation sides were higher than the pedicle screw-alone side (Figure 7A). In general, the maximum pull-out force on the augmentation side was significantly higher compared to the screw-only side (non-augmentation screws vs. augmentation screws: 288.5 ± 105.6 N vs. 401.1 ± 114.6 N) (Figure 7B). The maximal pull-out force was significantly increased by CaS/HA augmentation both in the thoracic (non-augmentation screws vs. augmentation screws: 278.8 ± 122.2 N vs. 359.5 ± 83.5 N) and lumbar (non-augmentation screws vs. augmentation screws: 311.8 ± 50.5 N vs. 500.8± 125.6 N) (Figure 7C,D). Overall, the maximum pull-out force of the augmented side was 39.0% higher than the pedicle screw-alone side (overall: 39.0%, thoracic: 28.9%, lumbar: 60.6%). The mechanical analysis of vertebral bone showed that the mean stiffness of the cement-augmented screw side was not statistically different to the screw-only side, including the thoracic and lumbar (Figure 7E–H).

**Figure 8:**
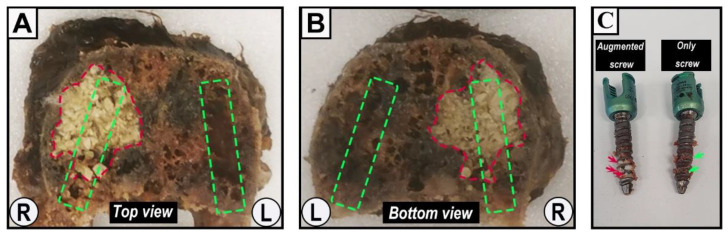
Representative specimens of vertebrae and fenestrated pedicle screws after pull-out testing. (**A**) The top section; and (**B**) the bottom section of vertebra L2 after axial cross-section along the midline of the pedicle. The red area of irregular shape indicates the CaS/HA cement-augmented area, while the rectangular area marked by the green dashed lines indicates the rough zone of the pedicle screw trajectory. “L” and “R” indicate the respective orientations. (**C**) A pair of pedicle screws (6.5/30 mm) from T1 after the pull-out experiment. Red arrows indicate remnants of CaS/HA cement; green arrows indicate remnants of the trabecular bone tissue.

Pedicle screw failure occurred at the screw-bone interface in both the screw-alone and the augmented screw sides. After testing, 10 specimens could be retrieved from the epoxy resin while the other vertebrae were damaged during removal of the osteoporotic vertebral body from the container due to the tight adhesion of the vertebral body and the epoxy resin. The vertebral cross-section photographs along the midline of the pedicle demonstrated that the cement was evenly distributed within the trabecular network in the vertebral body, which densified the loose architecture of the osteoporotic vertebral body. At the screw trajectory area, a local cavity was formed inside the vertebrae after screw extraction at the screw-only side; cement was still filled within the screw trajectory at the augmented side (Figure 8A,B). After testing, the threads of the augmented screws were filled with cement, but only little trabecular or bone marrow tissue filled the threads of the screws without augmentation (Figure 8C).

## 4. Discussion

In this study, we described the novel application of a recently established CaS/HA biomaterial cement for the augmentation of pedicle screws in the osteoporotic spine. By injecting CaS/HA cement into the internal channel of the fenestrated pedicle screws, the cement can be evenly distributed into the screw threads and into the surrounding trabecular in order to strengthen the interlocking effect at the bone-screw interface (Figure 5 and Figure 8). Biomechanical testing demonstrated that the described method of injectable CaS/HA cement improved the maximum pull-out force of fenestrated pedicle screws in osteoporotic vertebrae (Figure 7). The hypothesis that CaS/HA cement can enhance the interface between the pedicle screw and the osteoporotic bone and support the immediate anchoring of the pedicle screw in the osteoporotic vertebrae of the thoracic and lumbar spine was verified.

Anchoring of pedicle screws in the spine with poor bone quality remains challenging. The success of a pedicle screw instrumentation depends on the implant design as well as on the quality of the bone which could provide adequate compression between the threads and surrounding bone; this proves to be a critical aspect of fixation stability [30]. Previous studies found that there is a significant linear correlation between bone mineral density (BMD) and failure cycle and fatigue loads [31]; it is also suggested that for a BMD of thoracic and lumbar less than 80 mg/cm^3^, the stability of the pedicle screws may be insufficient so that additional stabilization should be considered [31]. Dual energy X-ray absorptiometry (DEXA) is the current “gold standard” for BMD measurement and osteoporosis screening. Osteoporosis is defined by the World Health Organization as ≥2.5 standard deviations below the mean BMD, as measured by DEXA [1]. However, DEXA measurements of BMD are affected by lumbar degenerative changes, scoliosis, compression fractures, and cannot distinguish between cortical and cancellous bone [26]. Some studies have found that the detection of BMD based on the measured HU value not only has a certain linear correlation with the BMD assessed by DEXA and quantitative computed tomography, but can also be used for the auxiliary diagnosis of osteoporosis by passing the above shortcomings of the DEXA method [26]. Two recent studies suggested that using HU for the diagnosis of osteoporosis aiming for high sensitivity may set a cutoff value of 150 HU, while aiming for a more balanced sensitivity-specificity ratio may require a cutoff value of 135 HU [29,32]. The HU of all vertebral bodies in this study met the diagnostic criteria for osteoporosis. In addition, DEXA can only measure the overall BMD of the vertebrae without the ability to determine the BMD of the screw trajectory. The HU value of the screw trajectory can be used for pedicle screw stability prediction [27]. There were no significant differences in the HU values of the trajectories nor in the potential augmentation area of the augmented-screw side and the contralateral area of the screw-only side. This consolidates that the difference in the pull-out force was exclusively mediated by the augmentation effect of the cement.

At present, pedicle screw augmentation techniques mainly include prefilling with cement prior to solid screw insertion and fenestrated pedicle screws injected with cement through the inner channel, but the biomechanical testing results of PMMA augmentation are controversial [33]. Compared to traditional solid pedicle screws, the extraction force of the fenestrated pedicle screw with PMMA augmentation is either increased, equal, or even decreased [33]. However, here the cannulated screw has the advantages of a shorter surgery time and a reduced risk of cement leakage [9,33]. In this study, the commercially available, fenestrated standard pedicle screws with the same size for each vertebra were used for CaS/HA cement augmentation. The optimal amount of cement used for proper screw fixation is also controversial and numerous studies on PMMA recommend 1 mL in the thoracic spine and 1.5–3 mL in the lumbar spine [5,16,33]. With reference to the amount of PMMA, 1 mL of CaS/HA cement in the thoracic spine and 2 mL in the lumbar spine were used in this study. The CaS/HA cement was evenly distributed in the anterior end of fenestrated pedicle screws both in the coronal and sagittal plane and it significantly enhanced the pull-out force of the pedicle screw without spinal canal leakage. The exposure of the CaS/HA cement in the endplate is due to the tight junction between the annulus fibrosus and the cartilage endplate or the marginal bone of the vertebral body resulting in damage of the cartilage endplate during specimen preparation. These results proved that the CaS/HA cement augmented fenestrated pedicle screw is an effective technique and confirmed that a dosing volume of 1 mL cement in the thoracic spine and 2 mL in the lumbar spine is effective. However, the biomechanical evaluation and comparison of screw implantation between the two techniques as well as the correlation of cement volume and mechanical properties is worth further investigation. The CaS/HA cement overall showed a 39% increase in the maximum pull-out force, which was lower than that of PMMA (50–100% [16,34]), but the increase in the lumbar vertebrae was 60.6% in this range, which may be related to vertebrae structure and bone cement amount. Due to the relatively small size of the thoracic vertebrae, the amount of cement that can be accommodated is limited, resulting in a limited increase in the pull-out resistance of the thoracic spine, which may also be related to the overall smaller force level of the thoracic spine [33]. The larger trabecular area of the lumbar vertebrae allows high doses of bone cement to be injected, which further leads to higher maximum pull-out forces. From a clinical point of view, the lumbar bears more stress than the thoracic because there is no sternum and ribs to share stress, and the higher pull-out resistance obtained may potentially reduce the risk of screw failure. The biomechanical relationship between cement volume and region needs to be further explored in another independent controlled trial. After the pull-out experiment, we observed that the CaS/HA cement was still evenly filled in the trabecular bone and between the threads of the screw, which confirms the good distribution and interdigitation ability of the CaS/HA cement [12]. This is another important reason why the cement enhances pull-out strength by filling the gap between the bony trabeculae and the thread of the screw in osteoporotic vertebrae [35]. This interface failure of CaS/HA cement occurs at the screw/bone interface, which is different from the failure modality of the PMMA composite/bone interface, indicating that the shear and tensile strength of CaS/HA, like calcium phosphate cement, are lower than those of cancellous bone [8,34].

Biphasic CaS/HA biomaterials have different rates of degradation, as the CaS phase is rapidly absorbed within 6–8 weeks in vivo while the HA phase persists for 9–12 months, integrating with new bone ingrowth over time [36]. The undesired degradation rates (too rapid or too slow) of single-phase degradable cement or the non-degradable nature of PMMA fail to match the bone ingrowth speed. Due to the lack of intrinsic osteoinductive cytokines, the functionalization of biomaterials with osteopromotive molecules is a promising option in bone tissue engineering [17], especially in the osteoporotic bone. Our previous studies have shown that CaS/HA materials can be used to locally co-deliver rhBMP-2 and zoledronic acid (ZA) to promote femoral defect repair [17] and bone regeneration in the osteoporotic femoral neck canal [37,38]. This is due to the fact that rhBMP-2 is physically entrapped in the resorbable CaS phase of the carrier for rapid release, whereas ZA is chemically bound to HA for long-term action [39]. Furthermore, Raina et al. showed that local co-delivery of rhBMP-2 and ZA by CaS/HA significantly enhanced screw anchoring [40]. Our recent data even indicates that the osteoporosis drug ZA seeks peri-implant HA particles and biologically activates them to induce significantly more new cancellous bone formation around the implant in an osteoporotic animal model of implant integration. All these data demonstrate that the suggested workflow is a promising option for the future usage of CaS/HA cement alone or after functionalization with osteo-regenerative molecules to enhance pedicle screw anchorage in patients with osteoporotic vertebrae. Further studies are warranted to elucidate whether this approach could aid in the reduction of pedicle screw failure and reduce the number of reoperations.

## 5. Limitations

Besides a successful outcome of the study, certain technical limitations need to be considered: the damaged cartilage endplate during specimen preparation due to the tight connection between the endplate and intervertebral disc in this study may affect the quantification of absolute pull-out force results. The anatomical variation of the pedicle in the available cadaveric vertebrae may also influence the biomechanical results. However, these limitations are not expected to affect a pairwise side-to-side comparison of individual vertebrae since specimen handling did not influence the morphology and macroscopic anatomy of the bone specimens [33]. Another limitation is that the axial pull-out test used in this study cannot fully simulate a physiological fatigue load situation, even though it is considered a standard test method for the fixing strength of screws [41]. The relationship between cement volume and biomechanics requires further exploration in another independent controlled trial. In addition, this experiment was a preliminary proof-of-concept study with a very limited sample size; a large sample size trial would be useful to further confirm our results.

## 6. Conclusions

This study demonstrates a novel application of the recently established injectable, biodegradable biphasic CaS/HA cement for augmentation of fenestrated pedicle screws in osteoporotic spine. In a cadaveric model, the CaS/HA augmentation was proven as an effective technique which significantly enhanced the required pull-out force of fenestrated pedicle screws in osteoporotic vertebrae in comparison with non-augmented screws. The technique allowed adequate cement distribution and interdigital ability to the surrounding trabecular bone. In addition, the CaS/HA material has been approved for clinical utilization, which could allow for an easier translation to the clinical application of this technique. In conclusion, this biodegradable biphasic CaS/HA cement could be a promising material for pedicle screw augmentation in the osteoporotic spine.

## Figures and Tables

**Table 1:** The individual, vertebra-specific pedicle screw sizes defined according to CT scan measurement results.

Location	Size (d/l) in mm	Location	Size (d/l) in mm	Location	Size (d/l) in mm
T1	6/30	T7	6/45	L1	7/50
T2	5/35	T8	6/50	L2	7/50
T3	5/40	T9	6/50	L3	7/50
T4	5/40	T10	7/45	L4	7/45
T5	5/45	T11	7/45	L5	7/40
T6	6/45	T12	7/50		

The screw sizes were defined by diameter “d” and length “l” (in mm).

## Data Availability

All data generated or analyzed during this study are included in this article.

## Supplementary Materials

The following supporting information can be downloaded at: https://www.mdpi.com/article/10.3390/jfb13040269/s1, Figure S1: CT scans of vertebrae with (A) outlier (T3) and (B-D) suboptimal pull-out test results (T1, T7, T11). “L” and “R” indicate the left and right side. The white rectangular areas indicate the potential cortical bone hyperplasia of the left pedicle in T1 and the potential microfracture areas inT3, T7 and T11.

## References

[1] Krenzlin H., Foelger A., Mailänder V., Blase C., Brockmann M., Düber C., Ringel F., Keric N. (2021). Novel Biodegradable Composite of Calcium Phosphate Cement and the Collagen I Mimetic P-15 for Pedicle Screw Augmentation in Osteoporotic Bone. Biomedicines.

[2] Reginster J.Y., Burlet N. (2006). Osteoporosis: A still increasing prevalence. Bone.

[3] Park S.B., Chung C.K. (2011). Strategies of spinal fusion on osteoporotic spine. J. Korean Neurosurg. Soc..

[4] Xie F., Zhou B., Wang J., Liu T., Wu X., Fang R., Kang Y., Dai R. (2018). Microstructural properties of trabecular bone autografts: Comparison of men and women with and without osteoporosis. Arch. Osteoporos..

[5] Paré P.E., Chappuis J.L., Rampersaud R., Agarwala A.O., Perra J.H., Erkan S., Wu C. (2011). Biomechanical evaluation of a novel fenestrated pedicle screw augmented with bone cement in osteoporotic spines. Spine (Phila Pa 1976).

[6] Abousayed M., Boktor J.G., Sultan A.M., Koptan W., El-Miligui Y. (2018). Augmentation of fenestrated pedicle screws with cement in patients with osteoporotic spine. J. Craniovertebr. Junction Spine.

[7] El Saman A., Meier S., Sander A., Kelm A., Marzi I., Laurer H. (2013). Reduced loosening rate and loss of correction following posterior stabilization with or without PMMA augmentation of pedicle screws in vertebral fractures in the elderly. Eur. J. Trauma Emerg. Surg..

[8] Tomé-Bermejo F., Piñera A.R., Alvarez-Galovich L. (2017). Osteoporosis and the Management of Spinal Degenerative Disease (I). Arch. Bone Jt. Surg..

[9] Shea T.M., Laun J., Gonzalez-Blohm S.A., Doulgeris J.J., Lee W.E., Aghayev K., Vrionis F.D. (2014). Designs and techniques that improve the pullout strength of pedicle screws in osteoporotic vertebrae: Current status. Biomed. Res. Int..

[10] Rometsch E., Spruit M., Zigler J.E., Menon V.K., Ouellet J.A., Mazel C., Härtl R., Espinoza K., Kandziora F. (2020). Screw-Related Complications after Instrumentation of the Osteoporotic Spine: A Systematic Literature Review with Meta-Analysis. Glob. Spine J..

[11] Sun H., Liu C., Liu H., Bai Y., Zhang Z., Li X., Li C., Yang H., Yang L. (2017). A novel injectable calcium phosphate-based nanocomposite for the augmentation of cannulated pedicle-screw fixation. Int. J. Nanomed..

[12] Sun H., Liu C., Li X., Liu H., Zhang W., Yang H., Li C., Yang L. (2020). A novel calcium phosphate-based nanocomposite for the augmentation of cement-injectable cannulated pedicle screws fixation: A cadaver and biomechanical study. J. Orthop. Translat..

[13] Frick C., Dietz A.C., Merritt K., Umbreit T.H., Tomazic-Jezic V.J. (2006). Effects of prosthetic materials on the host immune response: Evaluation of polymethyl-methacrylate (PMMA), polyethylene (PE), and polystyrene (PS) particles. J. Long Term Eff. Med. Implant..

[14] Rohmiller M.T., Schwalm D., Glattes R.C., Elalayli T.G., Spengler D.M. (2002). Evaluation of calcium sulfate paste for augmentation of lumbar pedicle screw pullout strength. Spine J..

[15] Cho W., Wu C., Erkan S., Kang M.M., Mehbod A.A., Transfeldt E.E. (2011). The effect on the pullout strength by the timing of pedicle screw insertion after calcium phosphate cement injection. J. Spinal Disord. Tech..

[16] Hoppe S., Keel M.J. (2017). Pedicle screw augmentation in osteoporotic spine: Indications, limitations and technical aspects. Eur. J. Trauma Emerg. Surg..

[17] Raina D.B., Matuszewski L.M., Vater C., Bolte J., Isaksson H., Lidgren L., Tägil M., Zwingenberger S. (2020). A facile one-stage treatment of critical bone defects using a calcium sulfate/hydroxyapatite biomaterial providing spatiotemporal delivery of bone morphogenic protein-2 and zoledronic acid. Sci. Adv..

[18] Alfotawi R., Naudi K., Dalby M.J., Tanner K.E., McMahon J.D., Ayoub A. (2013). Assessment of cellular viability on calcium sulphate/hydroxyapatite injectable scaffolds. J. Tissue Eng..

[19] Abramo A., Geijer M., Kopylov P., Tägil M. (2010). Osteotomy of distal radius fracture malunion using a fast remodeling bone substitute consisting of calcium sulphate and calcium phosphate. J. Biomed. Mater. Res. B Appl. Biomater..

[20] Nilsson M., Wielanek L., Wang J.S., Tanner K.E., Lidgren L. (2003). Factors influencing the compressive strength of an injectable calcium sulfate-hydroxyapatite cement. J. Mater. Sci. Mater. Med..

[21] Masala S., Nano G., Marcia S., Muto M., Fucci F.P., Simonetti G. (2012). Osteoporotic vertebral compression fractures augmentation by injectable partly resorbable ceramic bone substitute (Cerament™|SPINE SUPPORT): A prospective nonrandomized study. Neuroradiology.

[22] Marcia S., Boi C., Dragani M., Marini S., Marras M., Piras E., Anselmetti G.C., Masala S. (2012). Effectiveness of a bone substitute (CERAMENT™) as an alternative to PMMA in percutaneous vertebroplasty: 1-year follow-up on clinical outcome. Eur. Spine J..

[23] Kok J., Širka A., Liu Y., Tarasevičius Š., Belickas J., Tägil M., Lidgren L., Isaksson H., Raina D.B. (2021). Augmenting a dynamic hip screw with a calcium sulfate/hydroxyapatite biomaterial. Med. Eng. Phys..

[24] Zampelis V., Tägil M., Lidgren L., Isaksson H., Atroshi I., Wang J.S. (2013). The effect of a biphasic injectable bone substitute on the interface strength in a rabbit knee prosthesis model. J. Orthop. Surg. Res..

[25] Raina D.B., Markevičiūtė V., Stravinskas M., Kok J., Jacobson I., Liu Y., Sezgin E.A., Isaksson H., Zwingenberger S., Tägil M. (2022). A New Augmentation Method for Improved Screw Fixation in Fragile Bone. Front. Bioeng. Biotechnol..

[26] Li D., Sun C., Jiang J., Lu F., Xia X., Wang H., Zou F., Ma X. (2022). A study of screw placement to obtain the optimal pull-out resistance of lumbar pedicle screws-analysis of Hounsfield units measurements based on computed tomography. BMC Musculoskelet. Disord..

[27] Zhang R.J., Li H.M., Gao H., Jia C.Y., Xing T., Shen C.L. (2020). Associations between the hounsfield unit values of different trajectories and bone mineral density of vertebrae: Cortical bone and traditional trajectories. Am. J. Transl. Res..

[28] Kuhns C.A., Reiter M., Pfeiffer F., Choma T.J. (2014). Surgical strategies to improve fixation in the osteoporotic spine: The effects of tapping, cement augmentation, and screw trajectory. Global Spine J..

[29] Ahern D.P., McDonnell J.M., Riffault M., Evans S., Wagner S.C., Vaccaro A.R., Hoey D.A., Butler J.S. (2021). A meta-analysis of the diagnostic accuracy of Hounsfield units on computed topography relative to dual-energy X-ray absorptiometry for the diagnosis of osteoporosis in the spine surgery population. Spine J..

[30] Chevalier Y., Matsuura M., Krüger S., Traxler H., Fleege C., Rauschmann M., Schilling C. (2021). The effect of cement augmentation on pedicle screw fixation under various load cases: Results from a combined experimental, micro-CT, and micro-finite element analysis. Bone Jt. Res..

[31] Weiser L., Huber G., Sellenschloh K., Viezens L., Püschel K., Morlock M.M., Lehmann W. (2017). Insufficient stability of pedicle screws in osteoporotic vertebrae: Biomechanical correlation of bone mineral density and pedicle screw fixation strength. Eur Spine J..

[32] Zaidi Q., Danisa O.A., Cheng W. (2019). Measurement Techniques and Utility of Hounsfield Unit Values for Assessment of Bone Quality Prior to Spinal Instrumentation: A Review of Current Literature. Spine (Phila Pa 1976).

[33] Leichtle C.I., Lorenz A., Rothstock S., Happel J., Walter F., Shiozawa T., Leichtle U.G. (2016). Pull-out strength of cemented solid versus fenestrated pedicle screws in osteoporotic vertebrae. Bone Jt. Res..

[34] Elder B.D., Lo S.F., Holmes C., Goodwin C.R., Kosztowski T.A., Lina I.A., Locke J.E., Witham T.F. (2015). The biomechanics of pedicle screw augmentation with cement. Spine J..

[35] Son H.J., Choi S.H., Heo D.R., Kook I., Lee M.K., Ahn H.S., Kang C.N. (2021). Outcomes of the use of cement-augmented cannulated pedicle screws in lumbar spinal fusion. Spine J..

[36] Wang J.S., Tägil M., Isaksson H., Boström M., Lidgren L. (2016). Tissue reaction and material biodegradation of a calcium sulfate/apatite biphasic bone substitute in rat muscle. J. Orthop. Translat..

[37] Raina D.B., Širka A., Qayoom I., Teotia A.K., Liu Y., Tarasevicius S., Tanner K.E., Isaksson H., Kumar A., Tägil M. (2020). Long-Term Response to a Bioactive Biphasic Biomaterial in the Femoral Neck of Osteoporotic Rats. Tissue Eng. Part A.

[38] Širka A., Raina D.B., Isaksson H., Tanner K.E., Smailys A., Kumar A., Tarasevičius Š., Tägil M., Lidgren L. (2018). Calcium Sulphate/Hydroxyapatite Carrier for Bone Formation in the Femoral Neck of Osteoporotic Rats. Tissue Eng. Part A.

[39] Raina D.B., Isaksson H., Hettwer W., Kumar A., Lidgren L., Tägil M. (2016). A Biphasic Calcium Sulphate/Hydroxyapatite Carrier Containing Bone Morphogenic Protein-2 and Zoledronic Acid Generates Bone. Sci. Rep..

[40] Raina D.B., Larsson D., Sezgin E.A., Isaksson H., Tägil M., Lidgren L. (2019). Biomodulation of an implant for enhanced bone-implant anchorage. Acta Biomater..

[41] Kueny R.A., Kolb J.P., Lehmann W., Püschel K., Morlock M.M., Huber G. (2014). Influence of the screw augmentation technique and a diameter increase on pedicle screw fixation in the osteoporotic spine: Pullout versus fatigue testing. Eur. Spine J..

