# Supplementary material for: Evaluation of an Injectable Biphasic Calcium Sulfate/Hydroxyapatite Cement for the Augmentation of Fenestrated Pedicle Screws in Osteoporotic Vertebrae: A Biomechanical Cadaver Study
Source: J Funct Biomater. 2022 Dec 1;13(4):269. doi: 10.3390/jfb13040269 (PMC9786089; doi:10.3390/jfb13040269)
Supplement: Supplementary file 1 [file jfb-13-00269-s001.zip › jfb-2017945-supplementary.pdf]

Supplementary Materials:

Evaluation of an Injectable Biphasic Calcium Sulfate/ Hydroxyapatite Cement for the Augmentation of Fenestrated Pedicle Screws in Osteoporotic Vertebrae: A Biomechanical Cadaver Study

Xinggui Tian [1,2], Deepak B. Raina [3], Corina Vater [1,2], David Kilian [2], Tilman Ahlfeld [2], Ivan Platzek [4], Ute Nimtschke [5], Magnus Tägil [3], Lars Lidgren [3], Alexander Thomas [1], Uwe Platz [1], Klaus-Dieter Schaser [1], Alexander C. Disch [1] and Stefan Zwingenberger [1,2,*]

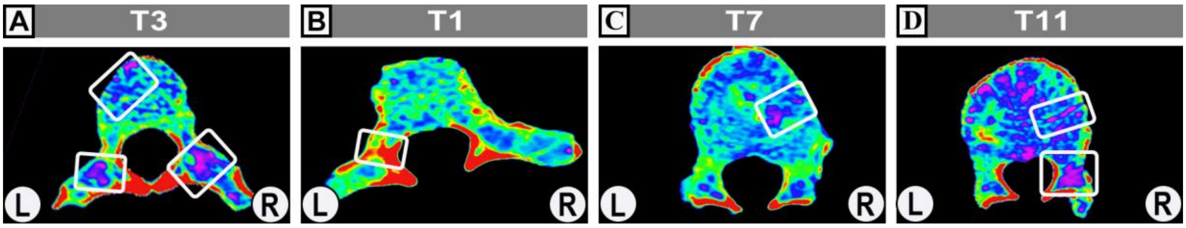

Figure S1. CT scans of vertebrae with (A) outlier (T3) and (B-D) suboptimal pull-out test results (T1, T7, T11). “L” and “R” indicate the left and right side. The white rectangular areas indicate the potential cortical bone hyperplasia of the left pedicle in T1 and the potential microfracture areas inT3, T7 and T11.
